# Assessing adherence to treatment guidelines and complications among atrial fibrillation patients in the United Arab Emirates

**DOI:** 10.3389/fcvm.2024.1359922

**Published:** 2024-07-10

**Authors:** Bayan Ayash, Diana Malaeb, Souheil Hallit, Hassan Hosseini

**Affiliations:** ^1^ College of Pharmacy, Gulf Medical University, Ajman, United Arab Emirates; ^2^ School of Medicine and Medical Sciences, Holy Spirit University of Kaslik, Jounieh, Lebanon; ^3^ Department of Psychology, College of Humanities, Effat University, Jeddah, Saudi Arabia; ^4^ Applied Science Research Center, Applied Science Private University, Amman, Jordan; ^5^ UPEC-University Paris-Est, Creteil, France; ^6^ RAMSAY SANTÉ, HPPE, Champigny sur Marne, France

**Keywords:** atrial fibrillation, treatment adherence, antithrombotic, complications, death, CHA_2_DS_2_-VASc, HAS-BLED

## Abstract

**Background:**

Atrial fibrillation (AF), a potential trigger for stroke development, is considered a modifiable condition that can halt complications, decrease mortality, and prevent morbidity. The CHA₂DS₂-VASc and HAS-BLED scores are categorized as risk assessment tools used to estimate the risk of thrombosis development and assess major bleeding among atrial fibrillation patients.

**Objectives:**

Our study aims to assess the adherence to post-discharge treatment recommendations according to CHA₂DS₂-VASc score risk group and evaluate the impact of CHA₂DS₂-VASc score and HAS-BLED score risk categories on death, length of hospital stay, complications, and hospital readmission among United Arab Emirates (UAE) patients.

**Methods:**

This was a multicenter retrospective study conducted from November 2022 to April 2023 in the United Arab Emirates. Medical charts for AF patients were assessed for possible enrolment in the study.

**Results:**

A total number of 400 patients were included with a mean age of 55 (±14.5) years. The majority were females (67.8%), and most had high CHA₂DS₂-VASc and HAS-BLED scores (60% and 57.3%, respectively). Our study showed that adherence to treatment recommendations upon discharge was 71.8%. The bivariate analysis showed that patients with a high CHA₂DS₂-VASc score had a significantly higher risk of death (*p*-value of 0.001), hospital readmission (*p*-value of 0.007), and complications (*p*-value of 0.044) vs. the low and moderate risk group with a *p*-value of <0.05. Furthermore, our findings showed that the risk of death (0.001), complications (0.057), and mean hospital stay (0.003) were significantly higher in the high HAS-BLED risk score compared to both the low- and moderate-risk categories. Hospital stay was significantly higher in CHA₂DS₂-VASc and HAS-BLED high-risk score categories compared to the low-risk score category with a *p*-value of <0.001.

**Conclusion:**

Our study concluded that the adherence to treatment guidelines in atrial fibrillation patients was high and showed that patients received the most effective and patient-centered treatment. In addition, our study concluded that the risk of complications and mortality was higher in high-risk category patients.

## Introduction

Atrial fibrillation (AF), an abnormal rhythm in the atria, is associated with significant morbidity and mortality (1). AF is considered the leading cause of stroke development (2) and is the most prevalent type of arrhythmia, affecting up to 33.5 million people worldwide with a prevalence of 2.5%–3.5% according to the Global Burden of Disease (3). Typical symptoms reported by patients include dyspnea, chest discomfort, dizziness, fatigue, and palpitations (4). The majority of the patients suffer from a bad quality of life manifested through depression, anxiety, and notable limitations in their social and physical activities (5). The treatment of AF encompasses treating hypertension, controlling hyperglycemia, and managing dyslipidemia.^1^ In patients with further risk factors for stroke development, oral anticoagulants (OACs) are considered an integral aspect of the management.

Non-adherence to the treatment recommendations can result in disease exacerbation and hospitalization. Multiple factors hinder compliance with the treatment recommendations including accurate assessment of the disease severity, requirement of regular follow-ups, comorbid conditions, hemodynamic stability status, and response to previous therapeutic regimens (6–8). Adherence to the treatment guidelines according to the CHA₂DS₂-VASc score is hindered due to the cost associated with the utilization of drugs, contraindications to medications, limited expertise in managing AF among healthcare professionals, poor access to certain medications, and loss to follow-up for further evaluation (9, 10).

According to the literature, despite recent advancements in AF treatment with the use of antithrombotics, the risk of recurrent hospitalization, cardiovascular complications, and death rate remain high (11, 12). Reasons for hospital readmission in AF are multifactorial and can include stroke development, bleeding progression, and concomitant disease exacerbation (13). Risk groups classified as intermediate and high CHA₂DS₂-VASc scores displayed elevated rates of comorbidities compared to those in the low-risk group, such as diabetes, hypertension, coronary heart disease (CHD), respiratory failure, hyperlipidemia, congestive heart failure (CHF), and valvular heart disease (14).

In the United Arab Emirates (UAE), the prevalence of cardiovascular disorders is high due to the increasing burden of established risk factors such as abdominal obesity, dyslipidemia, hypertension, and diabetes (15, 16). According to a previous study, the adherence to treatment guidelines was high among patients with comorbid uncontrolled diabetes and cardiovascular events; however, there is a need to assess adherence among cardiovascular patients with non-communicable diseases (17). In addition, studies conducted in Lebanon, Egypt, and Jordan assessed compliance with the guidelines among discharged patients, but there is limited data from the UAE (18–20). The study aimed to assess the adherence to treatment recommendations according to CHA₂DS₂-VASc score risk category and evaluate compliance to the management guidelines upon discharge from the hospital among AF patients. In addition, this study examined the correlation between the CHA₂DS₂-VASc score and HAS-BLED score risk categories on mortality rate, complication development, length of hospital stay, and hospital readmission status.

## Methods

### Study design and setting

**Figure 1:**
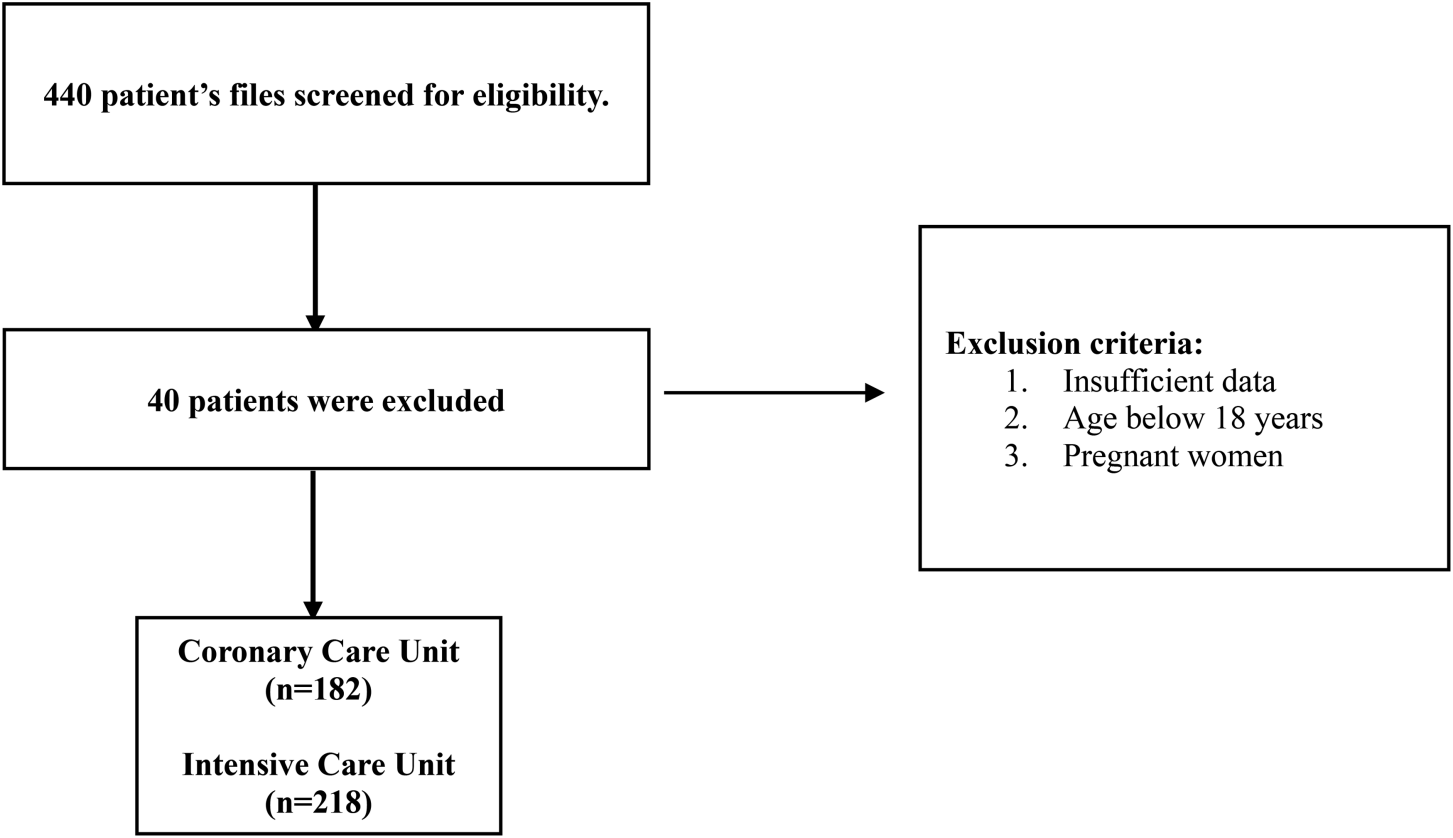
Operational flowchart.

This was a multicenter retrospective study conducted in the United Arab Emirates from November 2022 to April 2023. The medical records of AF patients admitted to the hospital between November 2015 and April 2023 were screened for enrolment in the study. A total of 440 medical charts were reviewed to assess the eligibility criteria, where 40 were excluded. The inclusion criteria included hospitalized adult patients with an AF diagnosis confirmed by a physician. The exclusion criteria included patients under 18 years of age, pregnancy, or medical charts with missing data (list of medical and medication history) that impede difficulty in calculating the HAS-BLED and CHA₂DS₂-VASc scores. Figure 1 shows a detailed study flowchart.

### Sample size calculation

The required sample size was calculated using Epi Info software, considering a confidence level of 95% and a risk of error of 5%. Given that adherence to atrial fibrillation treatment is 50%, a minimal sample size of 385 participants is needed (21).

### Study tool

The data collection process was performed by a registered pharmacist by identifying patients diagnosed with atrial fibrillation from the medical records. The data collection sheet was developed based on other studies conducted on AF patients (22–24).

The data collection sheet included different sections.

The first section gathered the sociodemographic and socioeconomic characteristics: age, gender, ethnic group, body mass index (BMI), drug or food allergy, cigarette smoking, length of hospital stays, and hospital care unit (coronary care unit, intensive care unit).

The second section included data about the type of atrial fibrillation (paroxysmal, persistent, permanent, and unknown) confirmed by a physician and other pertinent past medical history (hypertension, diabetes, renal/liver disease, dyslipidemia, stroke, vascular disease, congestive heart failure (CHF) and history of major bleeding.

The third section included an assessment of the CHA₂DS₂-VASc and HAS-BLED scores. The CHA₂DS₂-VASc score, a validated tool to assess the risk of stroke, was calculated based on the previous data collected from the medical records of patients using an online calculator.^2^ The CHA₂DS₂-VASc score was used to identify those who should receive anticoagulant treatment due to their significant mortality risk. This score assigns one point for factors such as congestive heart failure history with an ejection fraction of ≤40%, hypertension, diabetes mellitus, age >65 years, female sex, and history of vascular diseases (previous myocardial infarction, aortic plaque, or peripheral artery disease) and two points for patients with a history of stroke, thromboembolism, or transient ischemic attack and age ≥75 years. The maximum total score for CHA₂DS₂-VASc score is 9; a score of 0 indicates low risk, 1 is considered a moderate risk, and 2 and above is considered a high risk (25). According to treatment guidelines, if the CHA₂DS₂-VASc score is 0, it is advisable to abstain from antithrombotic treatment. For individuals with a CHA₂DS₂-VASc score of 1, it is recommended to consider antithrombotic therapy, with a preference for oral anticoagulation over antiplatelet therapy. If the CHA₂DS₂-VASc score is 2 or higher, it is strongly advised to start oral anticoagulation. Therefore, the data collection form assessed the adherence to treatment guidelines according to CHA₂DS₂-VASc score risk category, with specific reasons for non-adherence (absence of antiplatelets/anticoagulants or absence of anticoagulants) (26).

The HAS-BLED score is a clinical assessment tool used to evaluate the risk of bleeding in patients with atrial fibrillation (AF) who are on anticoagulant treatment, particularly oral anticoagulants such as warfarin or direct oral anticoagulants (DOACs) (27). It is calculated to estimate the risk of major bleeding in AF patients using an online calculator.^3^ This score assigns one point for the following factors: liver disease (cirrhosis or bilirubin >2× normal with AST/ALT/AP >3× normal), abnormal renal function (transplant, dialysis, Cr >2.26 mg/dl or 200 µmol/L), uncontrolled hypertension (>160 mmHg systolic), stroke history, previous major bleeding or predisposition to bleeding, unstable or high international normalized ratio (INR), elderly (age more than 65 years), concurrent use of alcohol, and use of medications that increase bleeding (such as antiplatelet and non-steroidal anti-inflammatory drugs) (28). The score ranges from 0 to 9, with a score of 0 indicating low risk, 1–2 suggesting moderate risk, and ≥3 indicating high risk for AF patients according to the HAS-BLED score. A HAS-BLED score of 3 or higher indicates that when prescribing oral anticoagulation, it's essential to take caution of bleeding occurrence (29).

The fourth section assessed adherence to non-communicable disease management upon discharge. The prescribed medications for non-communicable disease upon discharge were checked, and if any of these medications were missed, it was considered non-adherence to non-communicable disease treatment. The medications were antidiabetic, antihypertensive, antiplatelets, and lipid-lowering therapy with a specific reason for non-adherence (absence of antidiabetic, antihypertensive, antiplatelet, and lipid-lowering therapy).

The last section included an assessment of AF complications after hospital discharge such as cardiovascular events (cardiac arrest, myocardial infarction, bradycardia, cardiac thromboembolic event), genitourinary/gastrointestinal bleeding events, renal failure, and neurologic events (transient ischemic attack, stroke, peripheral nerve injury, phrenic nerve damage). In addition, hospital readmission status due to atrial fibrillation and death occurrence were documented as well.

### Statistical analysis

Data entry and analysis were performed on Statistical Package for the Social Sciences software (IBM SPSS Statistics, Version 23.0, IBM Corp., Armonk, NY, USA). The categorical variables were presented as infrequencies and percentages, and the continuous variables were presented as means with standard deviations (SDs). Statistical analysis was conducted using chi-square, Fisher’s exact test (used to compare between the three-category CHA₂DS₂-VASc score and HAS-BLED score and dichotomous or categorical variables), and analysis of variance (ANOVA) for continuous variables (length of hospital stay). Bonferroni *post hoc* tests were conducted on the groups. The statistical significance was set at a *p*-value of <0.05.

## Results

### Characteristics of study participants

A total number of 400 patients was enrolled in the study with a mean age (±standard deviation) of 55 (±14.5) years, the majority being females (67.8%) and from Arab countries (49%). The mean hospital stay was 1.25 ± 1.46 days, 54.5% were in the intensive care unit, and hypertension (62%) was the most common disease. The majority of participants (71.8%) reported adherence to non-communicable disease medication upon discharge. Most participants (82.5%) reported treatment adherence according to their CHA₂DS₂-VASc score risk category. All the sociodemographic characteristics are presented in Table 1.

**Table 1:** Sociodemographic characteristics and past medical history of respondents.

Characteristics	Frequency (%)
Age, mean (±standard deviation)	55 ± 14.5
Hospital stay, mean (±standard deviation)	1.25 ± 1.46
Gender	
• Male	129 (32.3)
• Female	271 (67.8)
Ethnic group	
• Arab	196 (49)
• Indian	46 (11.5)
• Pakistan	57 (14.3)
• United Arab Emirates	41 (10.3)
• Others	60 (15)
Hospital care unit	
• Coronary care unit	182 (45.5)
• Intensive care unit	218 (54.5)
Smoking	
• No	327 (81.8)
• Yes	73 (18.3)
Past medical history	
• Diabetes mellitus	158 (39.5)
• Hypertension	248 (62)
• Renal disease	47 (11.8)
• Liver disease	8 (2)
• Dyslipidemia	180 (45)
• Stroke/thromboembolism	17 (4.3)
• Vascular diseases	109 (27.3)
• Congestive heart failure	21 (5.3)
• Major bleeding	7 (1.8)
Atrial fibrillation diagnosis	
• Paroxysmal	114 (28.5)
• Persistent	36 (9)
• Permanent/long-standing	68 (17)
• Unknown/not mentioned	182 (45.5)
CHA₂DS₂-VASc score	
• Low risk (score of 0)	91 (22.8)
• Moderate risk (score of 1)	69 (17.2)
• High risk (score of >1)	240 (60)
HAS-BLED score	
• Low (score of 0)	68 (17)
• Moderate (score of 1–2)	103 (25.8)
• High (score of >3)	229 (57.3)
Adherence to non-communicable disease medication upon discharge	287 (71.8)
Reasons for non-adherence	
• Absence of antidiabetic	9 (2.3)
• Absence of antihypertensive	30 (7.5)
• Absence of antiplatelet	11 (2.8)
• Absence of statins	64 (16.1)
Treatment adherence according to CHA₂DS₂-VASc score risk category	330 (82.5)
If no, specify the reason	
• Absence of antiplatelet or anticoagulant	15 (3.8)
• Absence of anticoagulant	55 (13.8)

### CHA₂Ds₂-VASc and HAS-BLED scores

**Figure 2:**
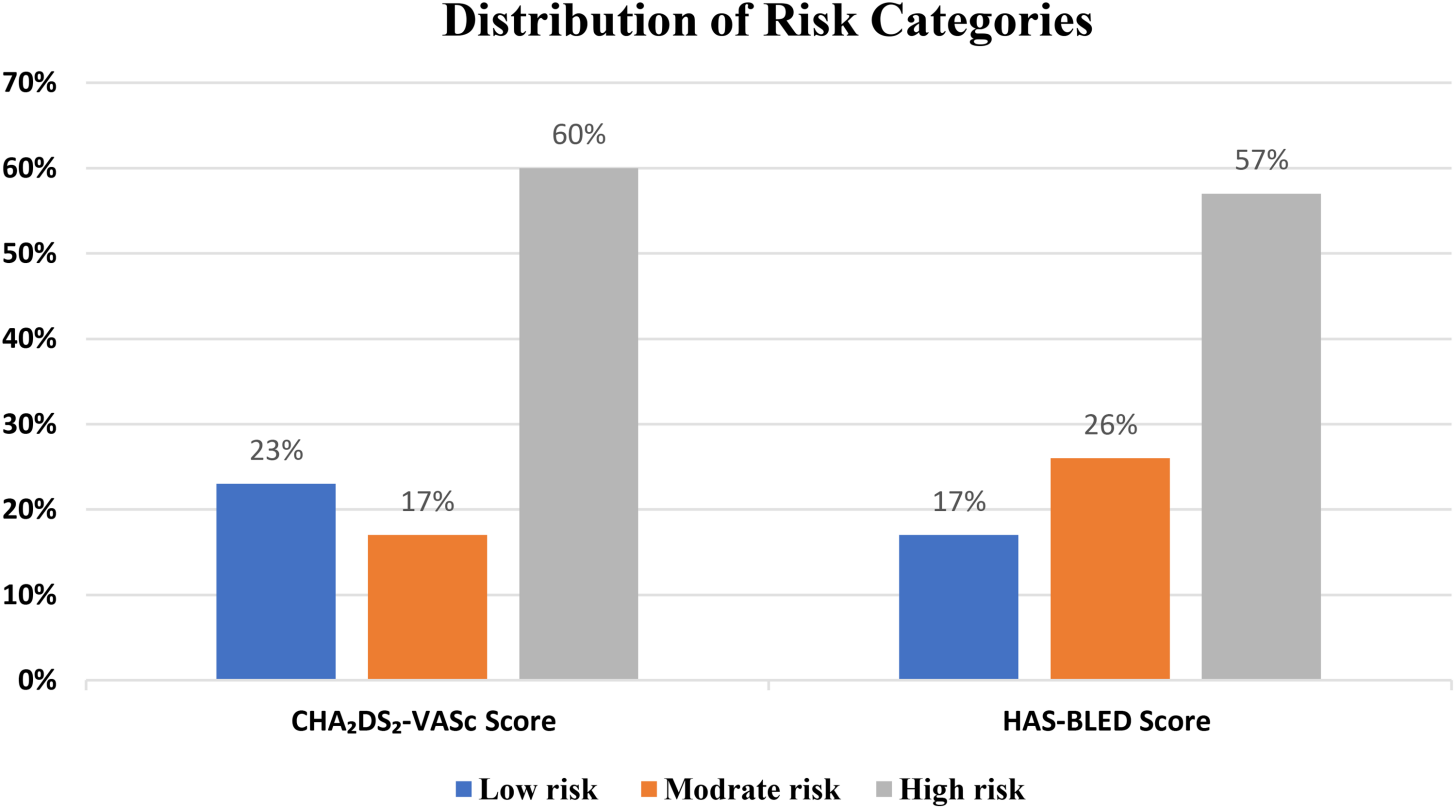
Distribution of CHA₂DS₂-VASc and HAS-BLED score risk categories among AF patients.

Most participants were classified as high risk (with scores of >1) based on the CHA₂DS₂-VASc scoring system (60%). Most of the patients had high bleeding risk (with scores of >3) based on the HAS-BLED scoring system (57.3%) (Figure 2).

### Bivariate analysis

#### Death

The bivariate analysis findings revealed a substantial increase in death occurrence among patients with a high CHA₂DS₂-VASc score when compared to those in the low- and moderate-risk categories, as indicated by a *p*-value of 0.001.

In terms of death in HAS-BLED score risk groups, the death occurrence was significantly higher in patients with high HAS-BLED scores compared to the low- and moderate-risk categories with a *p*-value of 0.001.

#### Complications

As for the complications, it was significantly higher in the CHA₂DS₂-VASc high-risk category compared to that in the low-risk category with a *p*-value of 0.044.

For the HAS-BLED score risk categories, 8.7% of the high-risk category had experienced complications compared to 2.9% of the low- and moderate-risk categories with a *p*-value of 0.057.

The data presented list several serious medical complications. Sepsis tops the list with the highest score of 6 (1.5), indicating that it is the most commonly noted complication. Both myocardial infarction and bleeding share the next level of significance with a score of 5 (1.3). Following these, stroke is recorded with a score of 4 (1), while cardiac thromboembolic events have a score of 3 (0.75), which places them lower on the scale of frequency. The complication rated as least frequent is the transient ischemic attack, with a score of 2 (0.5).

#### Hospital readmission

Bivariate analysis indicated that hospital readmission rates due to atrial fibrillation were higher in patients who had a high CHA₂DS₂-VASc score (23.1%) compared to those with low (11%) and moderate risk (10.1%) with a *p*-value of 0.007.

As for the HAS-BLED score, the bivariate analysis indicated that hospital readmission rates due to atrial fibrillation were statistically significantly higher in patients who had a high HAS-BLED score (23.3%) compared to those with moderate (7.8%) and low risk (16.2) with a *p*-value of 0.003 (Table 2).

**Table 2:** Correlation between the CHA₂DS₂-VASc score and HAS-BLED score risk categories and the outcomes of complications, death, and hospital readmission.

Variables	CHA₂DS₂-VASc score			*p*-value	HAS-BLED score			*p*-value
	Low risk	Moderate risk	High risk		Low risk	Moderate risk	High risk	
Death								
No	90 (98.9)	69 (100)	216 (93.7)	**0**.**001**	66 (97.1)	103 (100)	206 (90)	**0**.**001**
Yes	1 (1.1)	0 (0)	24 (6.3)	** **	2 (2.9)	0 (0)	23 (10)	** **
Complications								
No	89 (97.8)	67 (97.1)	219 (91.2)	**0**.**044**	66 (97.1)	100 (97.1)	209 (91.3)	**0**.**057**
Yes	2 (2.2)	2 (2.9)	21 (8.8)	** **	2 (2.9)	3 (2.9)	20 (8.7)	** **
Hospital readmission								
No	81 (89)	62 (89.9)	183 (76.9)	**0**.**007**	57 (83.8)	95 (92.2)	174 (76.7)	**0**.**003**
Yes	10 (11)	7 (10.1)	55 (23.1)		11 (16.2)	8 (7.8)	53 (23.3)	

Bold values mean that the results are significant.

#### Length of hospital stay

Our results showed that patients with high-risk CHA₂DS₂-VASc scores had a significantly higher mean length of hospital stay (1.54 ± 1.57) compared to those with both moderate and low risk (0.86 ± 1.12 and 0.81 ± 1.23) with a *p*-value of <0.001 and 0.010, respectively.

Our results showed that patients with both high and moderate-risk HAS-BLED scores had a significantly higher mean length of hospital stay (1.54 ± 1.55 and 1.16 ± 1.37) compared to low-risk patients (0. 44 ± 0.90) with a *p*-value of <0.001 and 0.002, respectively, as reflected in Table 3.

**Table 3:** Association between CHA₂DS₂-VASc and HAS-BLED scores on length of hospital stay.

Variables	Hospital stay Mean days ± standard deviation	Adjusted *p*-value
CHA₂DS₂-VASc score		
Low risk–moderate risk	0.81 ± 1.23–0.86 ± 1.12	1
Low risk–high risk	0.81 ± 1.23–1.54 ± 1.57	**<0.001**
Moderate risk–high risk	0.86 ± 1.12–1.54 ± 1.57	**0.010**
HAS-BLED score		
Low risk–moderate risk	0.44 ± 0.90–1.16 ± 1.37	**0.002**
Low risk–high risk	0.44 ± 0.90–1.54 ± 1.55	**<0.001**
Moderate risk–high risk	1.16 ± 1.37–1.54 ± 1.55	0.137

Bold values mean that the results are significant.

## Discussion

This study investigated the adherence to treatment guidelines according to CHA₂DS₂-VASc score risk categories and evaluated the adherence to discharge treatment recommendations. The study aimed to evaluate the impact of CHA₂DS₂-VASc score and HAS-BLED score risk categories on death, length of hospital stay, complications, and hospital readmission among United Arab Emirates patients. The results showed that patients with a moderate CHA₂DS₂-VASc score had a significantly higher risk of death, hospital readmission, and stroke complications than those in the low-risk group. Furthermore, our findings showed that the risk of death, stroke complications, and mean length of hospital stay were significantly higher in patients with high CHA₂DS₂-VASc risk scores compared to both low- and moderate-risk categories.

### Hospital stay

The results of the study revealed a significant association between both the CHA₂DS₂-VASc and HAS-BLED scores with the length of hospital stay in patients with atrial fibrillation. Higher CHA₂DS₂-VASc scores were associated with longer hospital stays, indicating that patients with a higher risk of stroke require more intensive medical management and monitoring during hospitalization. Similarly, higher HAS-BLED scores were also associated with longer hospital stays, which can be explained by the fact that patients with a high risk of bleeding may require additional medical attention and monitoring that extends the duration of hospitalization.

These findings are consistent with previous studies that have explored the association between risk scores and hospital outcomes in atrial fibrillation patients. According to a study conducted by Lip et al. (30), higher CHA₂DS₂-VASc scores were associated with an increased risk of stroke and mortality in atrial fibrillation patients.

Regarding the HAS-BLED score, previous research has shown a strong association between bleeding risk and clinical outcomes in atrial fibrillation patients. In a study done by Pisters et al. (28), higher HAS-BLED scores were associated with an increased risk of major bleeding events in AF patients treated with oral anticoagulants. In another study by Olesen et al. (31), higher HAS-BLED scores were associated with increased mortality in patients with atrial fibrillation.

### Treatment adherence according to CHA₂Ds₂-VASc score risk category

Our results showed that treatment according to CHA₂DS₂-VASc score risk category was highly adherent (82.5%) with the guidelines recommendations, which is higher than that in a previous study conducted in Italy where the adherence rate was reported to be 21.9% (32). According to Shore et al. (33), higher CHA₂DS₂-VASc scores were more likely to be prescribed oral anticoagulants than lower score categories.

It is extremely integral to document the other factors that should be considered while assessing the adherence to treatment according to CHA₂DS₂-VASc score categories as educational level, socioeconomic status, and cognitive function of the patients that play important roles in therapy (34).

The high adherence to treatment recommendations in our study can be interpreted by the fact that medical insurance with continuity of coverage and affordability of medications was accessible to most of the patients. Furthermore, physicians frequently follow the guidelines and recommendations by prescribing high-risk patients the suitable and most appropriate anticoagulant treatment. A previous study conducted by Proietti et al. (35) showed that around 25% of the patients with a high stroke risk (CHA₂DS₂-VASc score ≥2) were not prescribed anticoagulants (39%). The reasons for non-prescription included concerns about bleeding risks, patient refusal, and physician-related factors suggesting that treatment adherence remains a challenge in clinical practice.

### Adherence to disease management upon discharge

In this study, the results showed that adherence to non-communicable disease management upon discharge was 71.8%. No study was done that assessed the adherence to post-discharge medication; however, our study is a novel study that showed a high adherence. The United Arab Emirates boasts an effective healthcare system, evident in the decline of mortality rates and a substantial count of licensed healthcare providers. Additionally, employers are obligated to guarantee the provision of health insurance coverage for both their employees and their families (36).

### Death

The results of this study showed that the risk of death was significantly higher in patients who had a high CHA₂DS₂-VASc score compared to the moderate-risk category, which can be explained by the fact that hemodynamic instability triggered by atrial fibrillation raises the risk of death (37). Furthermore, the heart's ability to pump blood effectively is impaired by the fast and irregular beating associated with atrial fibrillation, which can result in decreased cardiac output and consequently organ dysfunction. In addition, Schnabel et al. (38) highlighted that hemodynamic instability influences mortality rates characterized by a greater risk of cardiovascular mortality, due to heart failure and sudden cardiac death. Numerous studies have shown that there is a link between AF and a higher risk of cardiovascular complications and all-cause deaths (39). The results of the later study are also consistent with the previous study of Friberg et al. (40), which demonstrated that higher HAS-BLED scores were associated with an increased risk of major bleeding and all-cause mortality in AF patients treated with oral anticoagulants. Similarly, a meta-analysis by Proietti et al. (2017) found that higher HAS-BLED scores were associated with a higher risk of major bleeding and cardiovascular mortality in AF patients (41).

### Complications

The results indicated that complications were significantly higher in patients with high CHA₂DS₂-VASc score risk category compared to the low-risk category consistent with the results of another study conducted by Lip et al. (30). Our findings can be explained that higher CHA₂DS₂-VASc scores reflect a higher risk of stroke due to an increase in the prevalence of the trigger factors such as congestive heart failure, hypertension, age, diabetes mellitus, stroke history, and vascular diseases. Furthermore, our findings showed that higher HAS-BLED scores are associated with an increase in complications, which can be interpreted by the fact that as the risk of bleeding increases, the HAS-BLED score increases, which may be associated with an increased risk of complications. A study by Chao et al. (42) (2018) evaluated that the CHA₂DS₂-VASc score is not only a predictor of stroke development but also serves as an indicator for future cardiovascular adverse outcomes in AF patients with and without anticoagulation therapy. The CHA₂DS₂-VASc score provides a comprehensive assessment of multiple risk factors, which enables clinicians to identify patients who may benefit from more intensive monitoring, preventive measures, and therapeutic interventions.

Recent evidence suggests that the CHA₂DS₂-VASc score might also predict thromboembolic events in non-AF populations, leading to a broader application of this risk assessment tool. The components of the CHA₂DS₂-VASc score could independently predict cardiovascular events in non-AF populations. The elements such as hypertension and age are significant predictors of cardiovascular risk irrespective of the presence of AF (43). A study showed a correlation between higher CHA₂DS₂-VASc scores and increased incidence of ischemic stroke in non-AF patients. It was demonstrated that the score's predictive value for ischemic events suggests a possible role in guiding primary prevention strategies (44). It was found that a higher CHA₂DS₂-VASc score in patients with specific conditions such as heart failure and peripheral artery disease, who do not have AF, correlates with a greater risk of thromboembolic complications, reinforcing the need for preventive measures (45).

The most recent study examined the predictive value of the CHA₂DS₂-VASc score for venous thromboembolism (VTE) in patients without AF. It confirmed that certain score components, especially age and previous VTE, were strongly predictive of new VTE events (46). The evidence from these studies supports a broader application of the CHA₂DS₂-VASc score for thromboembolic risk stratification in non-AF patients. This could lead to improved identification of high-risk individuals who might benefit from preventative therapies, such as anticoagulation, even in the absence of AF. Moreover, the score's components reflect a broad range of vascular risk factors, making it a useful tool for holistic cardiovascular risk assessment.

### Hospital readmission

Our findings showed that hospital readmission is significantly higher in patients with high-risk CHA₂DS₂-VASc scores compared to low and moderate risk, which is in line with the results of a cohort study conducted in the United States. The latter study showed an increase in the 30-day readmission from 7.2% for a CHA₂DS₂-VASc score of 0 to 24.6% for a score of ≥8 and 90-day readmission from 13.1% for a score of 0 to 38.5% for a score of 9 (47). This finding may be attributed to other factors that influence readmission, such as comorbidities, atrial fibrillation symptoms, and patient-related factors that were not specifically examined (48).

### Limitations

The present study comes with several limitations. The retrospective approach of the study might create underlying biases and restrict the identification of causal relationships. The data collected for this study is confined to the information documented in the medical records of patients. Relying only on the CHA₂DS₂-VASc score to assess the risk for complications development is not enough as it does not consider lifestyle variables or inflammatory biomarkers, which may increase the propensity for stroke development (49). Additionally, certain potential predictive factors, such as the status of the autonomic nervous system, were not considered.

## Conclusion

This study showed high adherence to treatment guidelines according to their CHA₂DS₂-VASc score risk category. Our study concluded that most of the participants were classified as high risk based on the CHA₂DS₂-VASc score and HAS-BLED score. Furthermore, our results highlighted that higher CHA₂DS₂-VASc and HAS-BLED scores were associated with an increased risk of death, complications, hospital readmission, and longer length of hospital stay. These findings highlight the importance of risk assessment in patients with atrial fibrillation, as both CHA₂DS₂-VASc and HAS-BLED scores were associated with various clinical outcomes. Tailored management and monitoring strategies based on these scores could potentially improve patient outcomes.

### Recommendations

Future research and studies with a bigger sample size are mandated to study and enhance the understanding of other atrial fibrillation risk factors and therapies that could influence the development of complications. Additionally, efforts to improve access to medications and enhance adherence to guidelines should be considered to optimize the management of non-communicable diseases. Our study showed the importance of raising awareness of treatment adherence and early identification of the risk factors among healthcare professionals and shed light on the implementation of a structured treatment approach.

## Data Availability

The original contributions presented in the study are included in the article/Supplementary Material, further inquiries can be directed to the corresponding author.

## Footnotes

^1^https://www.heart.org/en/health-topics/atrial-fibrillation/treatment-and-prevention-of-atrial-fibrillation/prevention-strategies-for-atrial-fibrillation-afib-or-af (accessed June 29, 2024).

^2^https://www.mdcalc.com/calc/801/cha2ds2-vasc-score-atrial-fibrillation-stroke-risk (accessed June 29, 2024).

^3^https://www.mdcalc.com/calc/807/has-bled-score-major-bleeding-risk (accessed June 29, 2024).

## References

[1] Li J Gao M Zhang M Liu D Li Z Du J Treatment of atrial fibrillation: a comprehensive review and practice guide. Cardiovasc J Afr. (2020) 31(3):153–8. 10.5830/CVJA-2019-064 32186324 PMC8762786

[2] Jame S Barnes G. Stroke and thromboembolism prevention in atrial fibrillation. Heart. (2020) 106(1):10–7. 10.1136/heartjnl-2019-314898 31533990 PMC7881355

[3] Sagris M Vardas EP Theofilis P Antonopoulos AS Oikonomou E Tousoulis D. Atrial fibrillation: pathogenesis, predisposing factors, and genetics. Int J Mol Sci. (2021) 23(1):6. 10.3390/ijms23010006 35008432 PMC8744894

[4] Heidt ST Kratz A Najarian K Hassett AL Oral H Gonzalez R Symptoms in atrial fibrillation: a contemporary review and future directions. J Atr Fibrillation. (2016) 9(1):1422. 10.4022/jafib.1422 27909518 PMC5089512

[5] Dorian P Ha AC. Symptoms in atrial fibrillation: patient and physician perspectives. Clin Electrophysiol. (2021) 7(5):575–7. 10.1016/j.jacep.2020.11.025 34016389

[6] Marzec LN Wang J Shah ND Chan PS Ting HH Gosch KL Influence of direct oral anticoagulants on rates of oral anticoagulation for atrial fibrillation. J Am Coll Cardiol. (2017) 69(20):2475–84. 10.1016/j.jacc.2017.03.540 28521884

[7] Bosch NA Cimini J Walkey AJ. Atrial fibrillation in the ICU. Chest. (2018) 154(6):1424–34. 10.1016/j.chest.2018.03.040 29627355 PMC6335260

[8] Choumane NS Malaeb DN Malaeb B Hallit S. A multicenter, prospective study evaluating the impact of the clinical pharmacist-physician counselling on warfarin therapy management in Lebanon. BMC Health Serv Res. (2018) 18:80. 10.1186/s12913-018-2874-7 29391010 PMC5796596

[9] Chaterji S Lian LG Lee TY Chua L Wee SY Yap SL Factors influencing primary care physicians’ prescribing behavior of anticoagulant therapy for the management of patients with non-valvular atrial fibrillation in Singapore: a qualitative research study. BMC Fam Pract. (2021) 22(1):1–1. 10.1186/s12875-021-01453-5 34034648 PMC8146184

[10] Malaeb D Hallit S Dia N Cherri S Maatouk I Nawas G Effects of sociodemographic and socioeconomic factors on stroke development in Lebanese patients with atrial fibrillation: a cross-sectional study. F1000Res. (2021) 10:793. 10.12688/f1000research.54236.2 34504688 PMC8383125

[11] Romiti GF Pastori D Rivera-Caravaca JM Ding WY Gue YX Menichelli D Adherence to the ‘atrial fibrillation better care’ pathway in patients with atrial fibrillation: impact on clinical outcomes—a systematic review and meta-analysis of 285,000 patients. Thromb Haemostasis. (2022) 122(03):406–14. 10.1055/a-1515-9630 34020488

[12] Kundu A O'Day K Shaikh AY Lessard DM Saczynski JS Yarzebski J Relation of atrial fibrillation in acute myocardial infarction to in-hospital complications and early hospital readmission. Am J Cardiol. (2016) 117(8):1213–8. 10.1016/j.amjcard.2016.01.012 26874548 PMC5075423

[13] Johnson BH Smoyer-Tomic KE Siu K Walker DR Sander S Huse D Readmission among hospitalized patients with nonvalvular atrial fibrillation. Am J Health-Syst Pharm. (2013) 70(5):414–22. 10.2146/ajhp120461 23413164

[14] Cheng L Kang S Lin L Wang H. The association between high CHA2DS2-VASc scores and short and long-term mortality for coronary care unit patients. Clin Appl Thromb Hemost. (2022) 28:10760296221117969. 10.1177/10760296221117969 35942685 PMC9373173

[15] Radaideh G Tzemos S Ali TM Eldershaby Y Joury J Abreu P. Cardiovascular risk factor burden in the United Arab Emirates (UAE): the Africa Middle East (AfME) Cardiovascular Epidemiological (ACE) study sub-analysis. Int Cardiovasc Forum J. (2017) 11:6–12. 10.17987/icfj.v11i0.414

[16] Oulhaj A Bakir S Aziz F Suliman A Almahmeed W Sourij H Agreement between cardiovascular disease risk assessment tools: an application to the United Arab Emirates population. PloS One. (2020) 15(1):e0228031. 10.1371/journal.pone.0228031 31978187 PMC6980489

[17] Alliabi FJA Jaber AAS Jallo MKI Baig MR. Adherence of physicians to evidence-based management guidelines for treating type 2 diabetes and atherosclerotic cardiovascular disease in Ajman, United Arab Emirates. BMC Prim Care. (2022) 23(1):70. 10.1186/s12875-022-01672-4 35392814 PMC8988318

[18] Malaeb D Cherri S Hallit S Saade S Hosseini H Salameh P. Assessment of post discharge medication prescription among Lebanese patients with cerebral infarction: results of a cross-sectional study. Clin Neurol Neurosurg. (2020) 191:105674. 10.1016/j.clineuro.2020.105674 31954365

[19] Kamal H Khodery M Elnady H Borai A Schaefer JH Fawi G Adherence to antithrombotic treatment and ischemic stroke recurrence in Egypt and Germany: a comparative analysis. Cerebrovasc Dis. (2021) 50(2):200–7. 10.1159/000512610 33477136

[20] Jarab AS Al-Qerem WA Hamam HW Alzoubi KH Abu Heshmeh SR Mukattash TL Medication adherence and its associated factors among outpatients with heart failure. Patient Prefer Adherence. (2023) 17:1209–20. 10.2147/PPA.S410371 37187575 PMC10178996

[21] Lowres N Giskes K Hespe C Freedman B. Reducing stroke risk in atrial fibrillation: adherence to guidelines has improved, but patient persistence with anticoagulant therapy remains suboptimal. Korean Circ J. (2019) 49(10):883–907. 10.4070/kcj.2019.0234 31535493 PMC6753021

[22] Krittayaphong R Winijkul A Kunjara-Na-Ayudhya R Apiyasawat S Siriwattana K Kanjanarutjawiwat W Adherence to anticoagulant guideline for atrial fibrillation improves outcomes in Asian population: the COOL-AF registry. Stroke. (2020)51(6):1772–80. 10.1161/STROKEAHA.120.029295 32390554

[23] Proietti M Lip GY Laroche C Fauchier L Marin F Nabauer M Relation of outcomes to ABC (atrial fibrillation better care) pathway adherent care in European patients with atrial fibrillation: an analysis from the ESC-EHRA EORP Atrial Fibrillation General Long-Term (AFGen LT) Registry. EP Europace. (2021) 23(2):174–83. 10.1093/europace/euaa274 33006613

[24] Guo Y Imberti JF Kotalczyk A Wang Y Lip GY. Atrial fibrillation better care pathway adherent care improves outcomes in Chinese patients with atrial fibrillation. JACC Asia. (2022) 2(4):422–9. 10.1016/j.jacasi.2022.01.007 36339366 PMC9627918

[25] Kim TH Yang PS Uhm JS Kim JY Pak HN Lee MH CHA2DS2-VASc Score (congestive heart failure, hypertension, age≥ 75 [doubled], diabetes mellitus, prior stroke or transient ischemic attack [doubled], vascular disease, age 65–74, female) for stroke in Asian patients with atrial fibrillation: a Korean nationwide sample cohort study. Stroke. (2017) 48(6):1524–30. 10.1161/STROKEAHA.117.016926 28455320

[26] January CT Wann LS Alpert JS Calkins H Cigarroa JE Cleveland JC 2014 AHA/ACC/HRS guideline for the management of patients with atrial fibrillation: a report of the American College of Cardiology/American Heart Association task force on practice guidelines and the Heart Rhythm Society. J Am Coll Cardiol. (2014) 64(21):e1–76. 10.1016/j.jacc.2014.03.022 24685669

[27] Methavigul K. Revised HAS-BLED score for bleeding prediction in atrial fibrillation patients with oral anticoagulants. J Arrhythm. (2022) 38(3):380–5. 10.1002/joa3.12709 35785373 PMC9237298

[28] Pisters R Lane DA Nieuwlaat R De Vos CB Crijns HJ Lip GY. A novel user-friendly score (HAS-BLED) to assess 1-year risk of major bleeding in patients with atrial fibrillation: the Euro Heart Survey. Chest. (2010) 138(5):1093–100. 10.1378/chest.10-0134 20299623

[29] Zhu W He W Guo L Wang X Hong K. The HAS-BLED score for predicting major bleeding risk in anticoagulated patients with atrial fibrillation: a systematic review and meta-analysis. Clin Cardiol. (2015) 38(9):555–61. 10.1002/clc.22435 26418409 PMC6490831

[30] Lip GY Nieuwlaat R Pisters R Lane DA Crijns HJ. Refining clinical risk stratification for predicting stroke and thromboembolism in atrial fibrillation using a novel risk factor-based approach: the euro heart survey on atrial fibrillation. Chest. (2010) 137(2):263–72. 10.1378/chest.09-1584 19762550

[31] Olesen JB Lip GY Kamper AL Hommel K Køber L Lane DA Stroke and bleeding in atrial fibrillation with chronic kidney disease. N Engl J Med. (2012) 367(7):625–35. 10.1056/NEJMoa1105594 22894575

[32] Giustozzi M Agnelli G Quattrocchi S Acciarresi M Alberti A Caso V Rates and determinants for the use of anticoagulation treatment before stroke in patients with known atrial fibrillation. Cerebrovasc Dis Extra. (2020) 10(2):44–9. 10.1159/000506923 32375143 PMC7250375

[33] Shore S Carey EP Turakhia MP Jackevicius CA Cunningham F Pilote L Adherence to dabigatran therapy and longitudinal patient outcomes: insights from the Veterans Health Administration. Am Heart J. (2014) 167(6):810–7. 10.1016/j.ahj.2014.03.023 24890529 PMC5381802

[34] Raparelli V Proietti M Cangemi R Lip GY Lane DA Basili S. Adherence to oral anticoagulant therapy in patients with atrial fibrillation. Thromb Haemostasis. (2017) 117(02):209–18. 10.1160/TH16-10-0757 27831592

[35] Proietti M Laroche C Opolski G Maggioni AP Boriani G Lip GY. AF Gen pilot investigators. ‘real-world’ atrial fibrillation management in Europe: observations from the 2-year follow-up of the EURObservational research programme-atrial fibrillation general registry pilot phase. Europace. (2017) 19(5):722–33. 10.1093/europace/euw112 27194538

[36] Koornneef EJ Robben PB Al Seiari MB Al Siksek Z. Health system reform in the emirate of Abu Dhabi, United Arab Emirates. Health Policy. (2012) 108(2-3):115–21. 10.1016/j.healthpol.2012.08.026 22998984

[37] Arrigo M Bettex D Rudiger A. Management of atrial fibrillation in critically ill patients. Crit Care Res Pract. (2014) 2024:840615. 10.1155/2014/840615 PMC3914350 24527212

[38] Schnabel RB Yin X Gona P Larson MG Beiser AS McManus DD 50 Year trends in atrial fibrillation prevalence, incidence, risk factors, and mortality in the Framingham Heart Study: a cohort study. Lancet. (2015) 386(9989):154–62. 10.1016/S0140-6736(14)61774-8 25960110 PMC4553037

[39] Ball J Carrington MJ McMurray JJ Stewart S. Atrial fibrillation: profile and burden of an evolving epidemic in the 21st century. Int J Cardiol. (2013) 167(5):1807–24. 10.1016/j.ijcard.2012.12.093 23380698

[40] Friberg L Rosenqvist M Lip GY. Evaluation of risk stratification schemes for ischaemic stroke and bleeding in 182 678 patients with atrial fibrillation: the Swedish atrial fibrillation cohort study. Eur Heart J. (2012) 33(12):1500–10. 10.1093/eurheartj/ehr488 22246443

[41] Lip GY Frison L Halperin JL Lane DA. Comparative validation of a novel risk score for predicting bleeding risk in anticoagulated patients with atrial fibrillation: the HAS-BLED (hypertension, abnormal renal/liver function, stroke, bleeding history or predisposition, labile INR, elderly, drugs/alcohol concomitantly) score. J Am Coll Cardiol. (2011) 57(2):173–80. 10.1016/j.jacc.2010.09.024 21111555

[42] Chao TF Liu CJ Wang KL Lin YJ Chang SL Lo LW Using the CHA2DS2-VASc score for refining stroke risk stratification in ‘low-risk’Asian patients with atrial fibrillation. J Am Coll Cardiol. (2014) 64(16):1658–65. 10.1016/j.jacc.2014.06.1203 25323252

[43] Paoletti Perini A Bartolini S Pieragnoli P Ricciardi G Perrotta L Valleggi A CHADS2 And CHA2DS2-VASc scores to predict morbidity and mortality in heart failure patients candidates to cardiac resynchronization therapy. Europace. (2014) 16(1):71–80. 10.1093/europace/eut190 23828875

[44] Kim KH Kim W Hwang SH Kang WY Cho SC Kim W The CHA2DS2VASc score can be used to stratify the prognosis of acute myocardial infarction patients irrespective of presence of atrial fibrillation. J Cardiol. (2015)65(2):121–7. 10.1016/j.jjcc.2014.04.011 24972564

[45] Peguero JG Issa O Podesta C Elmahdy HM Santana O Lamas GA. Usefulness of the CHA2DS2VASc score to predict postoperative stroke in patients having cardiac surgery independent of atrial fibrillation. Am J Cardiol. (2015) 115(6):758–62. 10.1016/j.amjcard.2014.12.037 25616533

[46] Sonaglioni A Lonati C Rigamonti E Vigano M Nicolosi GL Proietti M CHA2DS2-VASc Score stratifies mortality risk in heart failure patients aged 75 years and older with and without atrial fibrillation. Aging Clin Exp Res. (2022) 34(7):1707–20. 10.1007/s40520-022-02107-x 35294768 PMC8925288

[47] Lahewala S Arora S Patel P Kumar V Patel N Tripathi B Atrial fibrillation: utility of CHADS2 and CHA2DS2-VASc scores as predictors of readmission, mortality and resource utilization. Int J Cardiol. (2017) 245:162–7. 10.1016/j.ijcard.2017.06.090 28874288

[48] Tripathi B Atti V Kumar V Naraparaju V Sharma P Arora S Outcomes and resource utilization associated with readmissions after atrial fibrillation hospitalizations. J Am Heart Assoc. (2019) 8(19):e013026. 10.1161/JAHA.119.013026 31533511 PMC6806041

[49] Hijazi Z Aulin J Andersson U Alexander JH Gersh B Granger CB Biomarkers of inflammation and risk of cardiovascular events in anticoagulated patients with atrial fibrillation. Heart. (2016) 102(7):508–17. 10.1136/heartjnl-2015-308887 26839066

